# Computer-Interpretable Domain Knowledge for Drug-Induced Acute Kidney Injury: a Knowledge Graph Approach

**DOI:** 10.1038/s41597-026-07579-4

**Published:** 2026-06-10

**Authors:** Romy Vos, Iacopo Vagliano, Cornelis Boersma, Frank van Harmelen, Annette ten Teije, Joanna E. Klopotowska

**Affiliations:** 1 Department of Computer Science, Vrije Universiteit Amsterdam, Amsterdam, the Netherlands; 2 Department of Medical Informatics, Amsterdam UMC, University of Amsterdam, Amsterdam, the Netherlands; 3 Amsterdam Public Health Research Institute, Amsterdam, the Netherlands; 4 Department of Strategic Management, Open Universiteit, Heerlen, the Netherlands; 5 Health-Ecore, Zeist, the Netherlands; 6 Department of Health Sciences, UMC Groningen, Groningen, the Netherlands

## Abstract

In this study, a Knowledge Graph (KG) for Drug-Induced Acute Kidney Injury (DAKI) was developed to provide structured and standardized knowledge about drugs known to cause AKI. The DAKI-KG is integrated from several credible domain sources, which we standardized to international vocabularies. We demonstrate the applicability of the DAKI-KG through competency questions and expert evaluations. The envisioned two application areas are: (1) the KG can be used as input for a decision support tool to get insights into potential side-effects and drug-drug interactions harmful for kidneys, especially in patients with chronic kidney disease and (2) researchers can utilize the KG to derive confounders and variables for (causal) machine learning models or as input into link prediction methods. The evaluation results show the applicability of our KG through varying competency questions and use cases, though further improvements are needed before it is ready for clinical application. We anticipate the DAKI-KG to be relevant for tasks such as confounder identification, variable analysis, and the development of QA systems.

## Background & Summary

Acute Kidney Injury (AKI) is a critical health condition characterized by a rapid decline in kidney function, often associated with high morbidity, mortality, and increased healthcare costs^1^. Drug-induced AKI (DAKI) is a significant and often preventable cause of AKI with many commonly prescribed drugs associated with renal complications^2^. The detection and prevention of DAKI remains a challenging task^3^ given the lack of comprehensive tools to represent and access DAKI knowledge. Especially in patients with multi-morbidity, who use multiple medications simultaneously to manage their chronic diseases (i.e., polypharmacy).

The risk of DAKI is particularly high in patients with Chronic Kidney Disease (CKD)^4–6^. CKD impairs the kidneys’ ability to filter waste and maintain homeostasis, which makes the renal system more susceptible to additional stressors such as nephrotoxic drugs. Furthermore, the prevalence of polypharmacy in CKD patients increases the likelihood of adverse renal effects due to drug-drug interactions^7^. Given the delicate balance required in managing CKD patients, healthcare professionals face significant challenges in prescribing safe and effective treatments.

Knowledge Graphs (KGs) have emerged as powerful tools for structuring and representing complex information, enabling the integration, querying and inference of heterogeneous data^8^. A KG is a labeled, directed graph to organize knowledge using semantic relations and ontologies. Within the healthcare domain, KGs have been applied to several areas such as drug re-purposing, drug development and decision-support systems^9,10^. The potential of KGs lies in their ability to model complex relationships between data points, making it a natural way to model biological relations such as the pathophysiological mechanisms behind drug-drug interactions and drug side-effects. However, the application of KGs to domain-specific challenges such as DAKI is still in its infancy. Most existing (bio)medical KGs are broad in terms of content and often big, supporting data-driven prediction tasks. The size can hinder usability for both human and machine, and they are not tailored to a specific clinical context, which means they may have limited practical utility for healthcare professionals and researchers.

In this work, we focus on developing a domain-specific KG for DAKI, addressing the broader gap in representing and utilizing DAKI knowledge in healthcare. To maximize its practical value, we aligned our KG with the Dutch clinical setting. By structuring domain knowledge on drugs known to cause AKI, i.e., nephrotoxic drugs, this KG aims to serve as a robust resource for two main application areas: 1) the clinical context application area and 2) the research context application area. The first application area aims to support healthcare professionals in prescribing decisions by offering insight into known nephrotoxic side-effects and drug-drug interactions (DDIs). The second one aims to support clinical researchers in identifying relevant confounders when developing causal models for DAKI detection or prevention or for link prediction purposes.

Our contributions are: 1) an integration of trusted and well-established Dutch medical domain knowledge sources into a standardized KG; (2) the mapping of the the KG content to established international medical terminologies to ensure interoperability and international usability; (3) an evaluation of the KG for a knowledge-based query-answering system with standardized queries, specifically applied to the clinical or research context of DAKI in patients with CKD; and (4) a comprehensive overview of the challenges encountered when constructing the DAKI-KG to highlight key barriers, share insights, and guide future efforts in developing domain-specific knowledge graphs in healthcare.

## Related Works

To the best of our knowledge, we are the first to develop a DAKI-specific KG (DAKI-KG). We take inspiration from KG construction methods in other disease-specific KGs, where the content is centered on a single disease, as well as from more broadly oriented KGs in the biomedical and pharmaceutical domain.

### Disease-specific KGs

Wang *et al*.^11^ constructed a KG for diabetes complications and risk factors using expert-reviewed medical literature. They aimed to guide decision support around the treatment of diabetes by providing a risk score of diabetes complications. The construction process consisted of a manual process of literature review from databases such as PubMed and Embase, and data extraction conducted by experts.

The applicability of KG as a decision support tool can be seen in the work of Huang *et al*.^12^, who constructed a KG on depression and used several common data sources, such as PubMed, DrugBank^13^ and UMLS^14^. Natural language processing (NLP) tools were used when the concepts between sources did not align to create a standardized KG. Its applicability as a decision support tool was demonstrated through the use case of prescribing antidepressants.

Similarly, Sakor *et al*.^15^ assembled a KG to investigate adverse events caused by COVID-19 treatments. The content covers common data sources such as UMLS and DrugBank as well as SIDER^16^. They included drugs indicated for COVID-19 and its common comorbidities. As in the previous work, NLP tools were used to standardize free texts into triples. The authors demonstrated that this KG can be useful in inferring new DDIs and in discovering and exploring the context of adverse events.

### KGs for kidney diseases

Within the field of kidney-related diseases, Zhao *et al*.^17^ constructed a KG supporting Traditional Chinese Medicine (TCD) diagnosis and treatment of diabetic kidney disease. The knowledge is extracted from clinical guidelines and patient records, and manually organized into triples by domain-experts. The output of diagnostic queries over the KG were evaluated by experts and achieved promising results. Xie *et al*.^18^ also focused on TCD and developed a KG surrounding CKD diagnosis and TCD treatment. They extracted data from patient records and domain knowledge such as textbooks and TCM treatment guidelines. Data extraction was conducted through several NLP-based methods such as transformer-based and string similarity-based, combined with manual review. Kumar *et al*.^19^ utilized LLMs to generate KG triples in the context of CKD, based on relevant terms found in EHR-data of CKD patients. The paper further proposed a validation framework to assess the validity of LLM-derived KS. Finally, Man *et al*.^20^ investigated the development of a KG for kidney stone disease and integrated the PubMed literature with several public databases such as DrugBank. Relevant PubMed articles were identified through a structured search query, standardized using a semantic annotation tool, and subsequently mapped to multiple vocabularies such as UMLS and SNOMED CT^21^. This KG was evaluated in several use cases, showing its applicability to discover insights into kidney stone disease.

### Biomedical KGs

Broadly oriented KGs such as Hetionet^22^, BioKG^23^ and Oregano KG^24^ do not have a focus on a specific disease: rather, the idea is to create a large-scale KG to support computational tasks such as data-driven drug re-purposing. PrimeKG^25^ extends such an approach by integrating text-based descriptions to drug and disease entities, resulting in a multi-modal KG. Such works have shown to be promising in several task related to drug discovery^26,27^, although challenges remain, including incomplete or inaccurate data and limitations within link-prediction methods.

### Motivation

These works demonstrate the applicability of disease-specific KGs as well as generic biomedical KGs. These studies differ from our study in several ways: existing kidney-related KGs that are grounded in traditional Chinese medicine diverge in both data sources and primary objectives. Furthermore, LLM-derived KGs often lack clear data provenance and knowledge of the underlying sources. In this work, we focus specifically on DAKI, and prioritize Dutch sources that align with the prevailing medical guidelines and workflows, to ensure future applicability to Dutch clinical practice. In addition, the DAKI-KG assigned annotations to (1) drug-drug interactions, indicating whether they require an action and whether they are nephrotoxic, and (2) adverse events, specifying their frequency.

## Methods

### Primary Data Sources

Our DAKI-KG was developed using the databases and vocabularies presented in Table 1. These databases are known to be credible and align with the Dutch clinical practice. By standardizing data with internationally established vocabularies, we ensure that the DAKI-KG maintains international relevance.

**Table 1:** Primary data sources: FK (Farmacotherapeutisch Kompas, G-Standaard, SNOMED CT, ATC (Anatomical Therapeutic Chemical Classification System), MedDRA (Medical Dictionary for Regulatory Activities).

Name	Type	Country	Data Extracted
			- Indications
FK	Website	The Netherlands	– Contra-indications
			- Side-effects
**G-Standaard**	Database	The Netherlands	- Drug-Drug Interactions
**SNOMED CT**	Medical Terminology	International	- Standardized concepts for indications, contra-indications, side-effects
**ATC**	Classification System	International	- DrugClass- Standardized concepts for drugs
**MedDRA**	Medical Terminology	International	- Standardized Meddra Queries (SMQs) for CKD and AKI

#### Farmacotherapeutisch Kompas^28^

Farmacotherapeutisch Kompas (FK) is a Dutch medical reference website for healthcare professionals and patients, which guides the prescribing of medications. The primary source for the drug information in the FK is the Summary of Product Characteristics (SPC) text of drugs approved by the Dutch Medicines Evaluation Board and European Medicines Agency. In addition, sources such as Dutch medical guidelines are consulted. Other sources include widely recognized handbooks and medical literature. It is maintained by the Dutch Healthcare Institute, where an editorial team of pharmacists and physicians is responsible for compiling and maintaining the content. Scientific input is provided by a committee consisting of medical specialists and (clinical) pharmacists. FK contains information on all drugs that are authorized in the Netherlands, except very specific intramural drugs, such as radio-pharmaceuticals or tumor markers. The data format is semi-structured, with structured section headings filled with unstructured free text. Use of the FK for research purposes requires permission from the Dutch Healthcare Institute, which was granted for this study.

#### G-Standaard^29^

The G-Standaard is a comprehensive drug database used in the Netherlands to support healthcare providers, (hospital) pharmacies, and insurers in managing and dispensing drugs, medical devices, and related healthcare products. It is maintained by the Z-Index, an organization financed by contributions from healthcare organizations using the G-Standaard. The knowledge base component of the G-Standaard, such as the drug alerts, is developed and reviewed by multidisciplinary working groups of the Royal Dutch Pharmacists Association (KNMP in Dutch). These working groups consist of medical practitioners, including pharmacists, general practitioners, and medical specialists, who ensure the validity and currency of the content. Examples of such working groups include those for DDIs, contra-indications, pregnancy and lactation, and dosing according to renal impairment. The G-Standaard is not open source and requires a license; for this study, we obtained permission to use it. We used the G-Standaard version of February 2023.

#### SNOMED CT^21^

SNOMED CT is a comprehensive, multilingual, and structured medical terminology used in, among others, electronic health records to ensure consistent and accurate exchange of health information globally. It contains over 350,000 active concepts, which are ordered in a poly-hierarchy and can be described by multiple terms. SNOMED CT contains editions for different languages. SNOMED CT is licensed by SNOMED International and is freely available in member countries. We used the Dutch version from 31-10-24, retrieved through Nictiz, the Dutch National Release Center for SNOMED CT.

#### ATC Classification System^30^

Anatomical Therapeutic Chemical (ATC) Classification System is a globally recognized system for classifying drugs based on their therapeutic use, chemical properties, and mechanism of action. It is maintained by the World Health Organization (WHO) and is widely used in medical research, healthcare and regulatory practice. An ATC code is a unique code given to a medicine based on a hierarchical classification system and consists of five levels. The ATC system is distributed under terms that do not allow commercial use or any modification of the material.

#### MedDRA^31^

The Medical Dictionary for Regulatory Activities is a medical terminology, consisting of several levels forming a hierarchy. MedDRA encompasses terms related to diagnoses, adverse events and medical procedures. On top of the hierarchy, MedDRA defined *Standardized MedDRA Queries* (SMQs), which are groups of terms that define, contribute to, or cause a certain medical condition. We extracted MedDRA terms through the BioPortal API^32^ (https://bioportal.bioontology.org/). MedDRA requires a subscription, which we hold, and its terms do not allow for redistribution. Accordingly, no MedDRA terms are part of the DAKI-KG.

### The Data Extraction Process

**Fig. 1:**
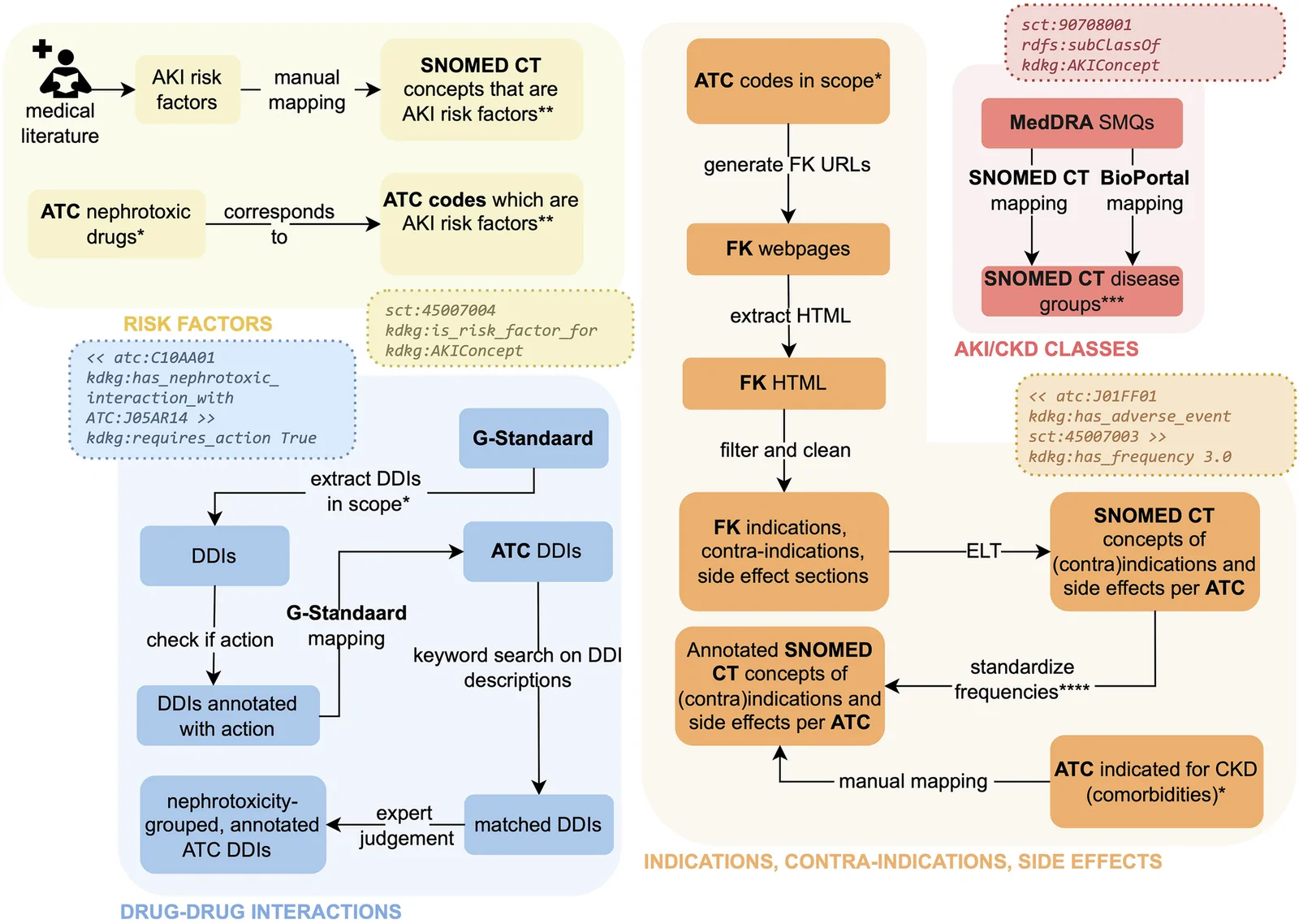
The data-extraction and standardization process for the DAKI-KG. *See Fig. 2 to see how we defined the scope of the DAKI-KG. **with *risk factor*, we refer to AKI risk factors. ***An additional manual expert was conducted for the AKI Class, as AKI is central in our KG. ****this step is only applicable to side effects.

The data extraction process is visualized in Fig. 1. We started with the selection of relevant drugs, which resulted in a list of ATC codes. Based on those codes, we retrieved the relevant pages from FK, from which we extracted data on contra-indications, indications and side effects. Then, we extracted all drug-drug interactions (DDIs) from the G-Standaard involving at least one relevant drug. Finally, we extracted a list of AKI risk factors from literature. We elaborate on this process in the subsections below.

#### Drugs

The main goal of our DAKI-KG was to provide a comprehensive overview of drugs known to cause AKI, with focus on patients with CKD and applicable in the Dutch clinical and research contexts. In the clinical context, the KG should allow the identification of nephrotoxic drugs to inform clinicians about drugs that could compromise kidney safety and provide safer alternatives for the kidneys if possible. In the research context, the KG should enable identification of nephrotoxic drugs but also drugs commonly prescribed in patients with CKD for CKD or other co-comorbidities. Therefore, we included the following drugs:

1. **Nephrotoxic drugs**. We used a comprehensive list proposed by Fernandez-Llaneza *et al*.^33^ to ensure that drugs for which experts agreed on their potential to cause AKI were included.
2. ** Drugs commonly used to treat CKD and frequent comorbidities in CKD patients**. We extracted lists of drugs from FK containing commonly indicated drugs for CKD and common comorbidities in patients with CKD: diabetes mellitus type 1 and 2, acute heart failure, chronic heart failure and hypertension.
3. **Drugs that interact with any of the above mentioned drugs**. This was based on the drugs included in the DDIs extracted from the G-Standaard. See the Drug-Drug Interactions section for the DDI extraction process.

**Fig. 2:**
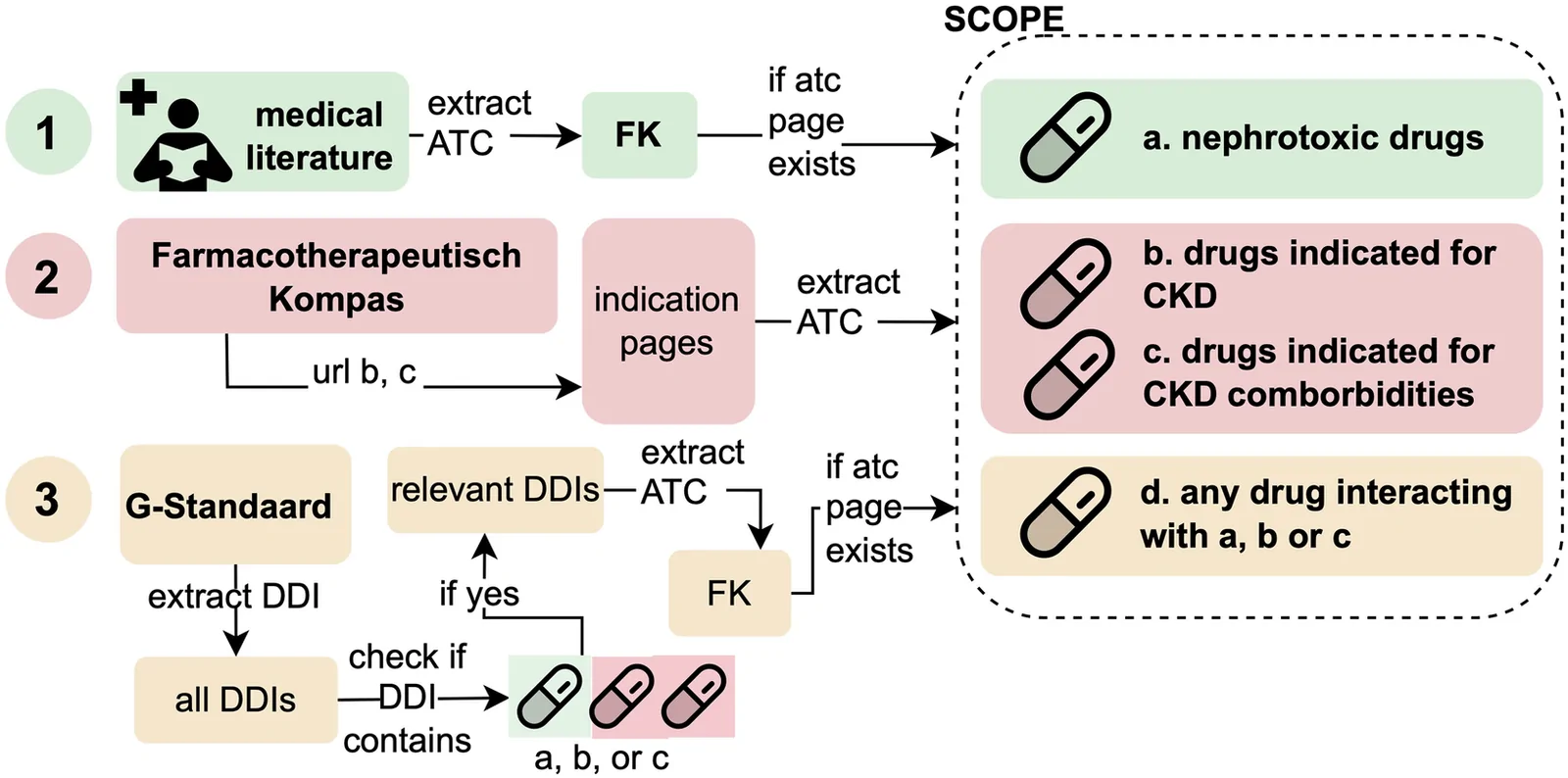
The scope and process of deriving the ATCs that are included in the DAKI-KG.

Note that in the work by Fernandez-Llaneza *et al*. and within the DDIs extracted from the G-Standaard, some drugs are involved that are not available in FK. To ensure that for all drugs included in the KG comprehensive data is provided, we excluded drugs not available in FK. The process of extracting the scope is visualized in Fig. 2.

#### Indications, Contra-Indications, Side-Effects

We used the ATC code to retrieve the FK web page for each drug of interest, by appending each ATC code to the FK base URL. When this triggered redirection pages due to multiple formulations or indications for one ATC code, we applied two criteria: for drugs identified as nephrotoxic, we selected only the specific page matching the administration route cited in Fernandez-Llaneza *et al*.^33^. For all other drugs, we included data from all redirected pages. After retrieving a page, we extracted the raw text from the indication section, the contra-indication section, and the side-effect section. We filtered out irrelevant information and applied a number of post-processing steps: we filtered out external references, subsection titles, and HTML tags. We replaced dosage-related “mg” with “milligram” to avoid ambiguity, and we captured side-effect frequencies with regular expressions.

With the content from the sections, we also kept the extraction date, ATC code and the title of the page. The same process was used for extracting indications, contra-indications and side-effects.

We used the BeautifulSoup^34^ package for the data extraction. All code and the exact URLs to retrieve data from FK can be found on our code repository (see Code Availability).

#### Drug-Drug Interactions

The DDIs from the G-Standaard are available in flowchart-based logic format enabling the implementation of DDI knowledge into clinical decision support systems. This flowchart-based logic takes into account patient and drug characteristics, has exclusion criteria, and includes logistical information if applicable. In this work, we were mainly interested in extracting (1) *Which two drugs interact*, (2) *whether this DDI was assessed as requiring an action at point-of-care*, and (3) *which DDIs are nephrotoxic*. To address step 1 and 2, we first extracted all DDIs and marked which require action. Subsequently, we included only DDIs that involve the drugs in scope of the DAKI-KG. For the drugs in scope for which no ATC match was retrieved in the G-Standaard, as a safeguard measure, we ran a keyword-based search on G-Standaard data. If we found an alternative ATC, we added this one. This was the case for only one drug (sirolimus). To address step 3, we extracted the background texts that are available in the G-Standaard for the majority of DDIs that require action. These background texts came in free text format. We further assessed these texts for nephrotoxicity as described in Data Standardization and Enrichment.

#### AKI Risk Factors

AKI risk factors may include comorbidities, patient characteristics, drugs, or comorbidities. We used an expert curated list with potential AKI risk factors, based on one international^35^ and one Dutch medical resource^36^.

### Data Standardization and Enrichment

#### ATC

We standardized all drug data to ATC codes. Since each FK page is linked to an ATC code (usually the specific 5th-level chemical substance), we use that exact code in the DAKI-KG. The G-Standaard works with a national generic product code (GPK in Dutch), which we mapped to ATC through the (many-to-one) mapping provided by the G-Standaard. We included only drugs and DDIs where both drugs within the DDI had a provided ATC code. To enable both precise and generic querying on drug groups (e.g, antibiotics), we linked each drug to its 2nd ATC level, representing pharmacological therapeutic subgroups.

#### SNOMED CT

The raw side-effects, indications and contra-indications texts from FK, the AKI risk factors terms, and the terms from the MedDRA SMQ for AKI and CKD were all converted to SNOMED CT concepts.

- **FK** Since the content on FK is in Dutch, we used an Entity Linking Tool (ELT) developed by Mohammadi *et al*.^37^. This NLP-based tool was developed to map content of Dutch clinical guidelines to SNOMED CT vocabulary. This tool selects sub-strings of the text, calculates a similarity score based on the overlap between the original string and the terms and synonyms in SNOMED CT, and the match is kept when this score is above a certain threshold. We ran each extracted FK paragraph (contra-indications, indications and side-effects) through the ELT separately. We slightly modified the tool: if there was an exact string match, we immediately included it and stopped searching for other matches. Otherwise, we searched through the other matched entities and kept the item with the highest similarity score and (if there were multiple) with the highest granularity (lower term in the SNOMED CT hierarchy). Here, we favored longer matches, ensuring the most granular match because the FK terms are often elaborate. We excluded matches with a similarity score below 0.5. Furthermore, we utilized the SNOMED CT hierarchy by only including matches that fall under the broad category *Clinical Finding* to ensure precision and to exclude noisy mappings. We also filtered out any mapping to the generic SNOMED CT concepts of *disease* (sct:64572001) and *adverse event* (sct:281647001).
  For each standardized term, the subsection title indicated the type of relation, while the page title and its ATC code specified the drug associated with that relation. For example, all terms listed under the *Side Effect* subsection on the Ibuprofen page were annotated as side effects of Ibuprofen.
  Drugs identified as indications for CKD and their comorbidities (see Fig. 2) were explicitly added to the KG as indications by manually mapping those comorbidity conditions to SNOMED CT concepts.
- **Risk Factors**. The risk factors were manually mapped to their relevant SNOMED CT identifiers.
- **MedDRA SMQs**. The SNOMED CT concepts following from the ELT are not standardized in terms of granularity. This hampers querying for higher-level disease groups, such as cardiovascular diseases or kidney diseases. Therefore, we included the full SNOMED CT hierarchy in our KG to enable querying over all descendants. However, using the hierarchy does not capture similar terms that exist in different branches. To circumvent this limitation of SNOMED CT hierarchy, we utilized MedDRA SMQs on top of standardized SNOMED CT concepts to create medical concept groups. Since the focus of DAKI-KG is (D)AKI, we utilized SMQs covering CKD and AKI. We mapped these from MedDRA to SNOMED through the Simple Mapping provided by SNOMED, and through the mappings via the BioPortal API^32^. Neither mapping method covered all terms. Therefore, we used both methods to maximize overall coverage. If the two methods provided different terms, we included both. For CKD, this was a fully automated process. Since AKI is the main focus of this paper, some manual steps were performed to ensure appropriate terms: (1) We did not start from all MedDRA AKI SMQ terms but utilized a selected version as described in^33^, (2) additional terms were included for MedDRA terms which did not result in any mapping automatically through a manual SNOMED CT search, and (3) after evaluation with the competency questions, additional terms were included to cover additionally relevant terms used in FK (polyuria (28442001), decreased renal function (76114004), abnormal renal function (39539005), abnormal urination (38671000119103), and retention of urine (267064002)). A medical expert (JEK) validated the mappings manually.

#### Side Effect Frequencies

Most side-effects extracted from FK have a frequency of occurrence. This information is of importance to clinicians and researchers when reasoning about nephrotoxic risks of drugs. Therefore, the side-effect frequencies were extracted together with the raw text from the side effect section in FK, but needed standardization as FK uses slightly varying terms for the frequency quantification. We mapped these variation to the following categories: very often (more than 10%), often (1–10%), sometimes (0,1–1%), rare (0,01-0,1%), very rare (less than 0,01%) and has also been reported (unclear frequency).

#### DDI Nephrotoxicity

The extracted background texts from the G-Standaard describe the mechanism of a drug-drug interaction. This is in free text format, hence we need to explicitly assess whether the extracted DDI background texts indicate any nephrotoxicity. To do so, we labeled DDIs as nephrotoxic using a keyword search on the texts. The keyword list was a manually created list of Dutch terms based on the MedDRA terms that fall under the MedDRA AKI SMQ (see our code repository for the full list of keywords). The texts that matched on one or more of the keywords were manually reviewed by RV, validated by JEK, and labeled as *“Not nephrotoxic”*, *“nephrotoxic”*, or *“risk factor for nephrotoxicity”*. To ensure that we did not miss any important nephrotoxic DDI, we checked with the following work^38^ and adjust in the case of any discrepancies.

### RDF-Graph Construction

The integrated KG is expressed in the Resource Description Framework (RDF) and serialized using the Turtle syntax^39^. RDF syntax models data through sets of object, predicate, subject triples, which together form an RDF graph. We serialized through RDF* (Turtle*), which allows for the annotation of individual triples (< < object, predicate, subject > > predicate annotation). This enabled us to enrich relation data with additional information such as labels over entire triples, metadata, and frequency values for side effects.

#### Entity roles

**Fig. 3:**
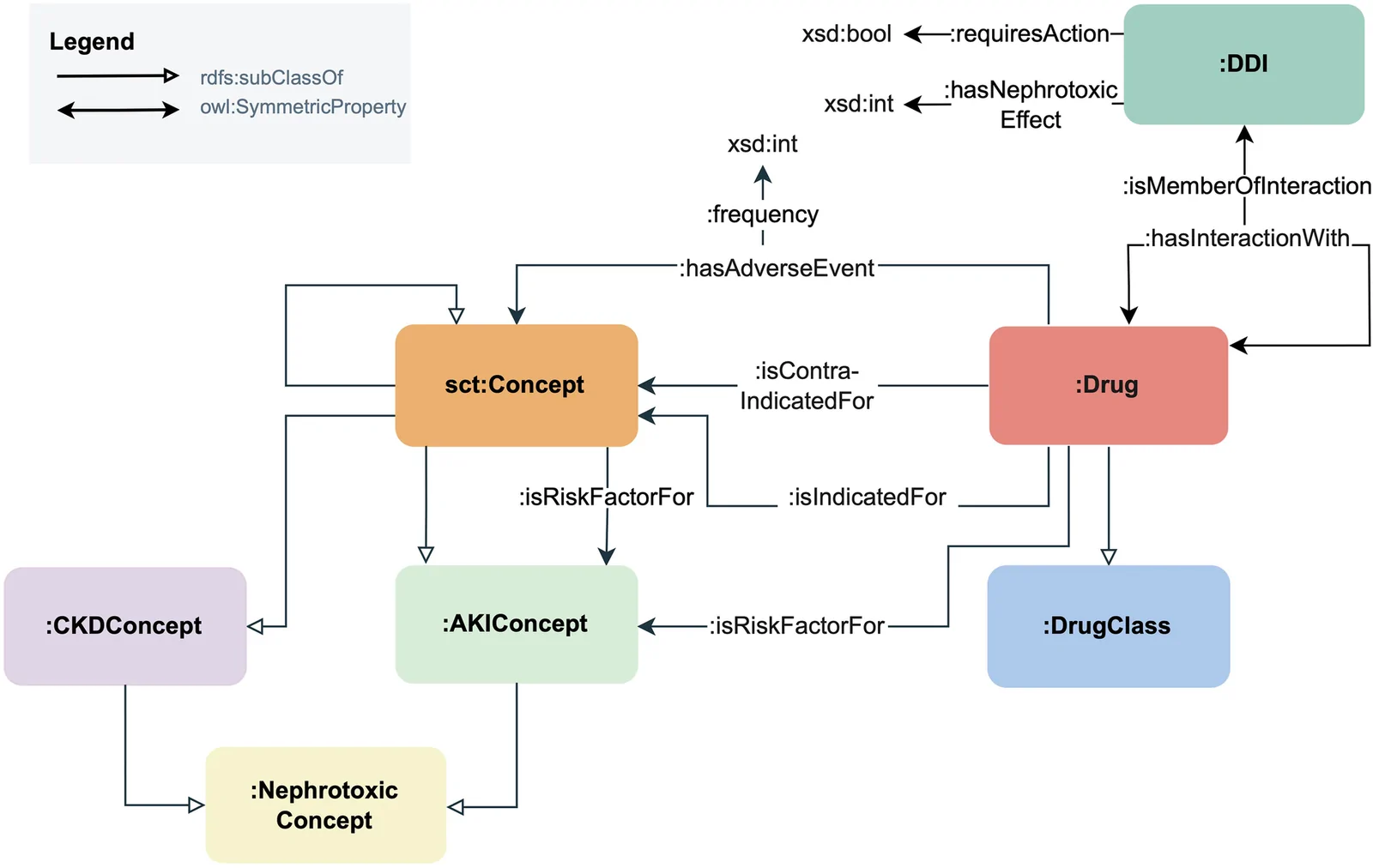
The DAKI-KG ontology. The default namespace refers to our own defined prefix, dakikg. An edge linked directly to another edge represents an RDF* annotation. The sct:Concept rdfs:subClassOf sct:Concept triples require an additional license (see Data Records).

To simplify integration and querying, we did not distinguish between entity types within the SNOMED CT terms, such as *Indication*, or *Adverse Event*: they were all represented simply as SNOMED CT concepts. Then, an entity like headache is represented uniformly, regardless of whether it occurs as a side-effect or as an indication. This modeling choice (as shown in Fig. 3) allows unified querying across contexts and supports analyses for different use cases.

#### Vocabularies

When no suitable match existed in established vocabularies, we created new properties in our own namespace (**dakikg**). See Table 2 for details. For hierarchical structures, such as the hierarchical relations in SNOMED CT entities (the *is_a* relation) and ATC hierarchies, we used the rdfs:subClassOf relations to preserve taxonomic information. Consequently, drugs and SNOMED CT entities are represented as classes rather than individual instances, to naturally represent their hierarchical structure.

**Table 2:** DAKI-KG properties and their descriptions. footnote*. These are RDF-star properties.

Property	Description
:hasAdverseEvent	Describes an adverse event of a drug.
:hasFrequency*	Describes for a drug-adverse event triple the reported frequency. The possible frequency categories are: 5 (very often), 4 (often), 3 (moderate), 2 (rare), 1 (very rare), and -1 (unspecified).
:isIndicatedFor	Describes a therapeutic use (indication) of a drug.
:isContraIndicatedFor	Describes a contraindication of a drug.
:isRiskFactorFor	Describes a risk factor for a disease. Both drugs and clinical findings can be a risk factor.
:hasInteractionWith	Describes an interaction between two drugs. This is a symmetric property.
:isMemberOfInteraction*	Describes the link from an interaction triple to an interaction type. RDF-star property.
:hasNephrotoxicEffect	Describes whether the effect of a drug-drug interaction is potentially nephrotoxic. The possible categories are: 0 (no nephrotoxic effect), 1 (DDI is considered higher risk in patients with diminished kidney function), and 2 (potentially nephrotoxic effect).
:requiresAction	Describes whether a drug-drug interaction requires any action. Boolean value.

#### Metadata

To ensure transparency and interoperability, we recorded essential descriptive elements. We captured the source of the data and the extraction date using properties from the Dublin Core Metadata Initiative vocabulary (dcterms)^40^. Labels are defined through the rdfs:label property and include both Dutch and English language labels, indicated with a language tag. For SNOMED CT entities, we denoted the SNOMED CT Preferred Terms (PT) through a skos:prefLabel^41^ predicate, with one PT provided in English and one in Dutch. Additionally, we added metadata related to the ELT: the similarity score and the FK source fragment on which the mapping was based. Note that high, or even perfect similarity scores are not always an indication of correctness.

#### Graph Database

We stored the RDF in a GraphDB triple store and queried via SPARQL queries, enabling flexible retrieval and integration with other semantic datasets. All query results in this work are retrieved using GraphDB’s rdfsplus-optimized ruleset.

## Data Records

The DAKI-KG can be downloaded from Zenodo^42^. Parts of the graph can also be separately downloaded. We ensured each separate file is self-contained: the graphs are organized by main source but remain fully compatible, allowing for easy integration as well as separation if desired. The files are available in RDF-star (ttls) format.

The DAKI-KG consists of different named graphs: The *ontology* file describes the classes and properties defined within the dakikg namespace. The *intrinsic_drug_properties* file contains the indications, contra-indications and side-effects. The *drug_interactions* file contains the drug-drug interactions, the *aki_risk_factors* file contains drugs and concepts that are AKI risk factors, and *nephrotoxic_classes* contains the concepts that fall under the AKI or CKD class.

The *snomed_ct_hierarchy* and *snomed_ct_pt* files represent the SNOMED CT hierarchy and preferred terms, respectively: these files are not publicly available through the repository due to licensing restrictions. The DAKI-KG can be used without those files, but we strongly recommend to include the SNOMED CT hierarchy file (resulting in the sct:Concept rdfs:subClassOf sct:Concept triples) to retrieve the benefits of hierarchical querying. Code and instructions for this can be found in our repository.

Finally, we have graphs describing metadata properties: (1) *elt_metadata*, containing all triples derived by the ELT, the similarity score from the ELT, the isolated fragment the ELT matched upon, and the extraction date, and (2) *drug_inclusion_rationales*, describing the reason for inclusion for the main drugs of focus (a, b and c of Fig. 2).

## Data Overview

An overview of the entity and relation types in the DAKI-KG is shown in Fig. 3. The final DAKI-KG contains 785 unique ATCs (drugs) and 4753 actively connected SNOMED CT concepts. The full SNOMED CT hierarchy contains 382,278 concepts, which are not used in the DAKI-KG except for their rdfs:subClassOf predicate during querying. Furthermore, the two SMQ-derived medical concept groups, AKIConcept and CKDConcept, have respectively 1634 and 6168 indirect members, and 42 and 199 direct members. The DAKI-KG contains 66 drug classes, with 12 members on average per class. In total 960 DDI types were included, with 519 identified as requiring an action. We identified 76 nephrotoxic DDIs (direct and indirect), and 30 DDIs where reduced kidney function is mentioned as a risk factor. The number of edges for different triple types are shown in Table 3.

**Table 3:** The average outgoing degree (AvgOutgoing) is calculated by counting all triples of a certain subject type, predicate type, and object type, and dividing by the total number of distinct subjects.

Subject	Predicate	Object	AvgOutgoing	AvgInverse	TotalEdges
:Drug	:hasInteractionWith	:Drug	54.74	54.74	42971
:Drug	:hasAdverseEvent	sct:Concept	47.97	7.92	37659
:Drug	:isIndicatedFor	sct:Concept	5.05	0.83	3966
:Drug	:isContraIndicatedFor	sct:Concept	4.50	0.74	3535
:Drug	:isRiskFactorFor	:NephrotoxicClass	0.07	55	55
sct:Concept	:isRiskFactorFor	:NephrotoxicClass	0.01	53	53

For the average inverse degree (AvgInverse), we divided by the total number of distinct objects. For the SNOMED CT concepts (sct:Concepts), we only counted concepts that are part of the graph itself, rather than the full SNOMED CT hierarchy (concepts with at least one relationship that is not rdfs:subClassOf). The predicate hasInteractionWith is symmetric, which has been inferred before counting.

**Fig. 4:**
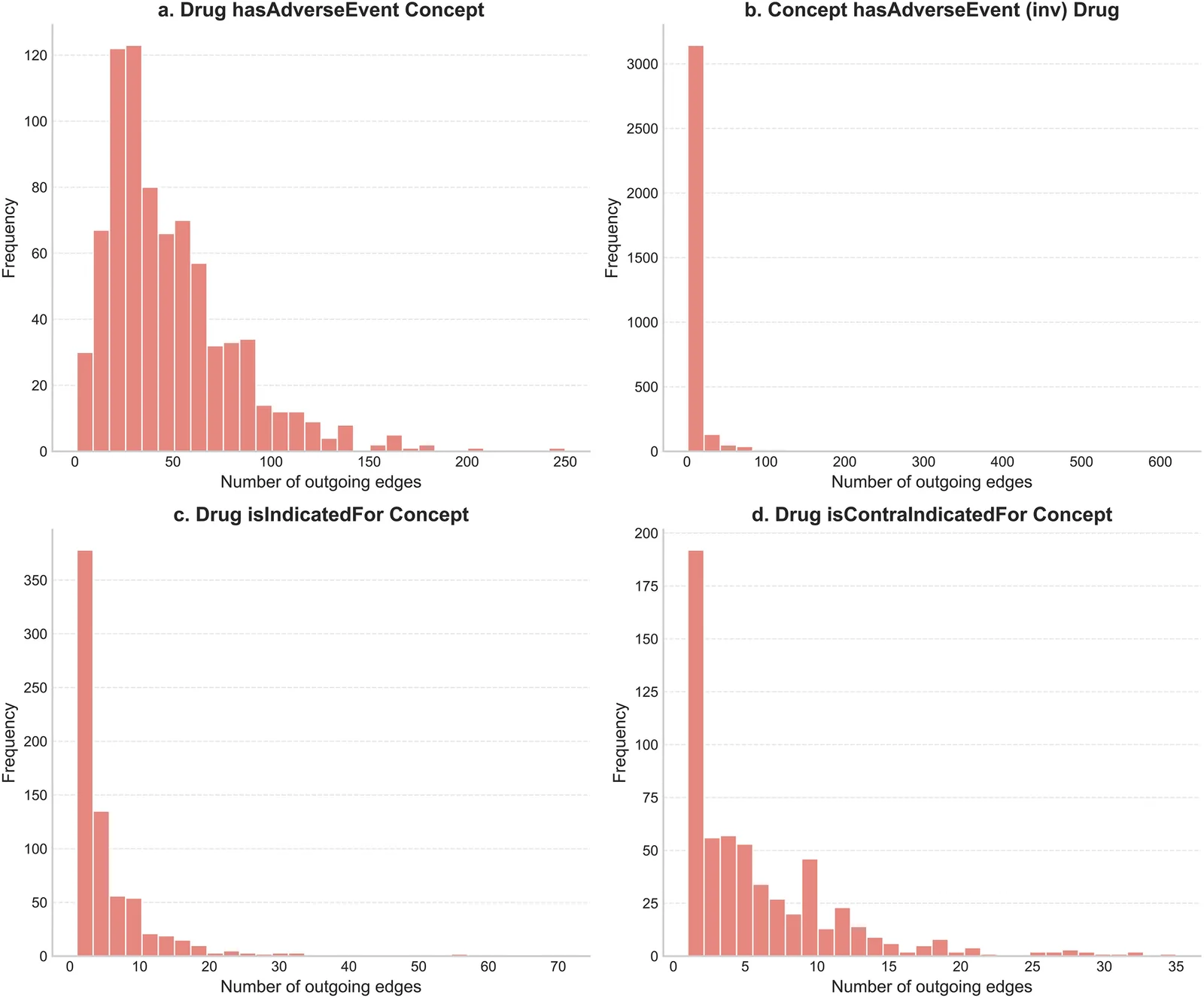
Degree distributions. Panels (**a**), (**c**) and (**d**) show the number of SNOMED CT concepts per drug for those involved in each relation type. Panel (**b**) shows the inverse: the number of associated drugs per adverse event, indicating side-effect prevalence.

Figure 4 displays the degree-distribution for side-effects, indications and contra-indications. Most drugs included in DAKI-KG have approximately 20–40 side-effects, with some outliers that have over 150. On the other hand, most side-effects are linked to very few drugs, implying that many of the side-effects are rare. Furthermore, most drugs are linked to one or a few (contra)indications.

## Technical Validation

We evaluated the DAKI-KG on the level of entity correctness (for the automated mappings from FK using ELT), and as a whole when all sources were integrated in DAKI-KG. The latter was done through two sets of competency questions (CQs), focused on (1) the clinical context application area and (2) the research context application area.

### Entity Linking Tool

To evaluate mapping quality of the ELT, we performed an expert evaluation with two medical experts (JEK, CB). At the time of ELT evaluation, we randomly sampled 1.25% of all extracted paragraphs from the FK. This equaled to 528 mappings. The full-text fragments from FK served as the gold standard. We did not take into account whether the terms in the full-text fragments existed in SNOMED CT or not, to emphasize overall clinical usefulness rather than solely compliance to SNOMED CT. Each paragraph length could contain any number of (golden standard) mappings. The mappings were evaluated on **precision**, i.e. *how many of the given mappings were mapped to a fitting SNOMED concept* and **recall**, i.e. *how many relevant concepts were retrieved from the golden standard*.

#### Precision

To calculate the precision, we gave the set of paragraphs and their mappings to the two medical experts. They were asked to evaluate if the mappings provided by the automated mapping tool were correct, incorrect, or partially correct, in the context of its FK subsection (e.g., indication or side-effects).

#### Recall

To calculate the recall, we considered each gold standard fragment individually and verified whether there was an overlapping mapping that was judged as correct by the medical experts. An overlapping mapping refers to a fuzzy match between a SNOMED CT-mapped concept and any portion of a corresponding concept in the gold standard. This additional matching step was required since the ELT operates on complete paragraphs rather than the segmented gold standard terms. Therefore, it may not produce exact fragment-level mappings. With the mappings matched to the gold standard, we used the expert-judgements (correct, partially correct and incorrect scored 1, 0.5 and 0, respectively) to calculate the final metrics. If the experts judged multiple mappings as correct for a single gold-standard fragment, we counted all of them as true positives and increased the length of the gold standard accordingly to avoid inflating recall: the expert judgement overrules the golden standard in such cases. Following Mohammadi *et al*.^37^, we also measured Recall*, where we only considered concepts that have a matching term in SNOMED CT, such that the ELT is being fairly evaluated for its task (mapping concepts to SNOMED CT, which naturally restricts the mapping to concepts present in SNOMED CT).

#### Inter-rater agreement

The inter-rater agreement between the two medical experts was calculated using the quadratic Cohen’s kappa score. This score properly accounts for the ordinal nature of the response categories: a disagreement between *Correct* and *Incorrect* is considered more severe than one between *Correct* and *Partially Correct*.

#### ELT Validation Results

We found the ELT to have an average precision of 0.80, and an average recall and recall* of 0.67 and 0.76, respectively. See Table 4 for the breakdown per entity type. The inter-rater agreement was 0.68, indicating substantial agreement^43^. The identified disagreements were mainly rooted in conflicting views on which level of granularity is acceptable. We distinguish the following precision errors: **(1) spurious span**, where the ELT mapped something to a part of the sentence that is not part of a gold standard fragment, i.e. *a frequent side effect is hypertension* is mapped to *side effect*. Then, **(2) incorrect addition**, where the ELT selected an incorrect term by matching the wrong fragment (e.g., linking *diabetes type 1* to *sialidosis type 1* based on *type 1*), leading to a fully incorrect mapping. Thirdly, **(3) under-specification**, if the ELT found a perfect match for a part of the gold standard, even though a more precise match existed which was not selected because of a lower similarity score, i.e. *diabetes type 1* was mapped to *diabetes* rather than to *diabetes mellitus type 1*. In some of these cases, the gold standard term did not exist in SNOMED CT. This particulaly occurred when mapping (contra)indications terms where complex phrases are used, i.e., *Maintenance treatment of schizophrenia in adults who have been sufficiently stabilized with oral olanzapine*. Finally, **(4) entity conflation**, where the ELT found a match for a wrongly selected sentence fragment, i.e., the last word of one sentence with the first word of the next sentence, or two comma-separated terms that describe different concepts (*nausea, vomiting* should be mapped to *nausea* and *vomiting* separately, but the ELT merged them.

**Table 4:** Scores over different mapping types.

	Precision	Recall	Recall*	n
**indications**	0.67	0.75	0.91	78
**contra-indications**	0.63	0.48	0.62	30
**side effects**	0.84	0.68	0.75	420
**all**	0.80	0.67	0.76	528

The distribution of the error types is shown in Table 5. The vast majority of the errors was due to *under-specification*. This is also due to limitations within SNOMED CT, where the gold standard terms do not exist in SNOMED CT. This also holds for the *incorrect addition*, where an unfit mapping (with a seemingly random addition) is possibly selected because the ideal fragment simply does not exist in SNOMED CT. Additionally, during evaluation of the CQs, we also found a case of an ignored negation leading to a false positive match.

**Table 5:** Distribution of error categories observed during the ELT assessment.

Error Category	Proportion	Count
Spurious span	17%	29
Incorrect addition	26%	45
Under-specification	51%	87
Entity conflation	6%	11

### Competency Questions

The set of CQ was defined in collaboration with a medical expert (JEK). The CQs consisted of a question and a set of correct answers in the form of drugs, DDI names, or concept names. A subset of the final CQs is shown in Table 6. The final set comprises 12 research CQs and 12 clinical CQs, including two overlapping pairs where a research and clinical CQ yield identical queries and answers despite different phrasing. The full list can be found on our repository, as well as the queries used to answer them.

**Table 6:** Examples of competency questions and their application area.

ID	Question	Application area
Q1.3	Which drugs for the treatment of heart failure have AKI as a side effect?	Clinical
Q1.8	Which drugs for the treatment of diabetes are contra-indicated in patients with CKD?	Clinical
Q1.11	Which side-effects are very frequent (10%) for lithium?	Clinical
Q2.4	Which drugs with AKI as side-effect are contra-indicated in patients with ckd?	Research
Q2.11	I am studying the association between metamizol and AKI, which AKI risk factors should I consider?	Research

#### Hierarchical Querying

We manually wrote SPARQL queries to answer the CQs. Specific entities (drugs or diseases) that were mentioned in the CQs were manually mapped to ATC codes and SNOMED CT concepts, by simply searching the exact term and finding the closest match. We also queried for disease-descendants where relevant (e.g., if a generic *diabetes* was mentioned in the query, we queried for *diabetes* and its more specific descendants). In some queries (*CQ 1.10 What side effects may occur when prescribing naproxen, furosemide, and enalapril concomitantly?*) this is not as straightforward: to find the side effects that occur across multiple drugs (which may present an accumulated risk), simply retrieving all obvious overlapping concepts will miss related side effects: terms in the source data are sometimes worded differently, which can lead to different SNOMED CT mappings for the same cumulative risk which is then missed by such queries. Querying for the descendants is more complex in polypharmacy, where multiple drugs have different hierarchies and there is no clear “main" category. To resolve this, we used a SPARQL query that identifies the drug whose side effect sits highest in the SNOMED CT hierarchy and then counts how many descendant concepts from that branch appear among the other drugs. This allowed us to group related terms (e.g., grouping vertigo under dizziness), increasing the identified shared side effects from 13 to 25 in the case of *CQ1.10*. For queries surrounding the terms AKI and CKD, we utilized the predefined AKI and CKD groupings from the DAKI-KG (as well as their descendants), which grouped related concepts even when they come from different SNOMED CT branches.

#### Metrics

The quantitative results of the CQs were obtained by simply counting the number of correct answers in the query output. The expert set of answers was not intended to be exhaustive: hence our CQ evaluation focuses on recall.

Furthermore, we divided the CQ-evaluation in three categories: (1) CQs that were fully answered, (2) CQs that were partially answered, and (3) CQs that were not answered. For the CQs that could not (fully) be answered, we investigated the error causes and provide suggestions on how such CQs could be answered in future optimized versions of the DAKI-KG.

#### CQ Validation Results

The evaluation results based on CQs are shown in Table 7. We identify several error types. First, errors caused by the **ELT**. This was the main cause of error, and is further described in the ELT Validation Results section.

**Table 7:** Error analysis for the CQs. Note that one CQ can contain multiple error causes. The score column depicts the total score, based on the exact number of correctly recalled answers per CQ.

	Cause of Error	Research	Clinical
**Fully correct CQs**	**N/A**	2.5, 2.7 (2)	1.4, 1.9, 1.12 (3)
**Partially correct CQs**	**ELT**	2.2, 2.4, 2.6, 2.9, 2.10 (5)	1.2, 1.3, 1.10, 1.11 (4)
	**Grouping**	2.1, 2.3, 2.4, 2.6, 2.8 (5)	1.1, 1.10 (2)
	**GS drug(s) OOS**	2.4 (1)	1.7 (1)
	**GS content OOS**	2.6, 2.12 (2)	1.8 (1)
	**GS content OOD**	—	1.5, 1.6 (2)
**Incorrect CQs**	**ELT**	—	—
	**Grouping**	—	—
	**GS drug(s) OOS**	—	—
	**GS content OOS**	2.11 (1)	—
	**GS content OOD**	—	—
**Score (Recall/Recall-OOS)**		65%/75%	75%/85%

Another error type is caused by **(lack of) grouping between related concepts**. More precisely: the DAKI-KG fails to retrieve certain relevant golden standard (FK) terms because the SNOMED CT concepts used in the query do not encompass the full range of related terms; the gold-standard term may exist in SNOMED CT, but lies outside the hierarchy of the queried concept. This reflects a limitation in the structure of SNOMED CT and hence the mappings in the context of the DAKI-KG: similar terms may not share the same concept or hierarchical branch, making effective grouping difficult. For example, *liver impairment* is not located under the same SNOMED branch as *liver failure*, even though it should ideally be included in the results when searching for side-effects related to liver.

One way to address this limitation is to query not only by concept identifiers but also by concept labels (i.e., retrieving all concepts that include the term “liver”). Note that for the CQ evaluation, we focused on querying the obvious disease explicitly mentioned in the CQ itself (*liver disease*) and its descendants rather than a text-based search. For AKI and CKD-related queries which are the main topics of the DAKI-KG, we utilized the MedDRA-based groupings to overcome this problem. We have seen that the addition of those groupings was extremely beneficial for querying the DAKI-KG.

However, as we depended on MedDRA-to-SNOMED CT mappings while converting the MedDRA groupings, some SNOMED CT terms may still be overlooked, despite the use of two different mapping methods and a manual validation of the AKI SMQ. After evaluation, we added some additional terms to the AKIConcept class, resulting in an improved AKIConcept class. Note that we did not update the evaluation scores to ensure fairness.

Finally, we defined three out-of-scope (OOS) errors (errors because the golden standard data was never given to the KG): **OOS-d,** **OOS-c** and **OOS-r**. The **OOS-d** error refers to cases where a **drug** is included in the gold standard (here FK), but is not retrievable as the drug is not in scope of the DAKI-KG (as defined in Fig. 2). The **OOS-c** error refers to cases where the right answers were not retrieved because the **content** was present in a section of FK that was not in scope to be extracted for the DAKI-KG. More specifically, there were cases where a kidney-related risk was only mentioned in the warnings section (out-of-scope for the DAKI-KG) rather than in the contra-indications section. Furthermore, the **OOS-r** errors are due to content which has been changed or **revised** since the version used in the DAKI-KG. In future versions of the DAKI-KG, we plan to extend the number of drugs and/or content to cover such cases.

**Fig. 5:**
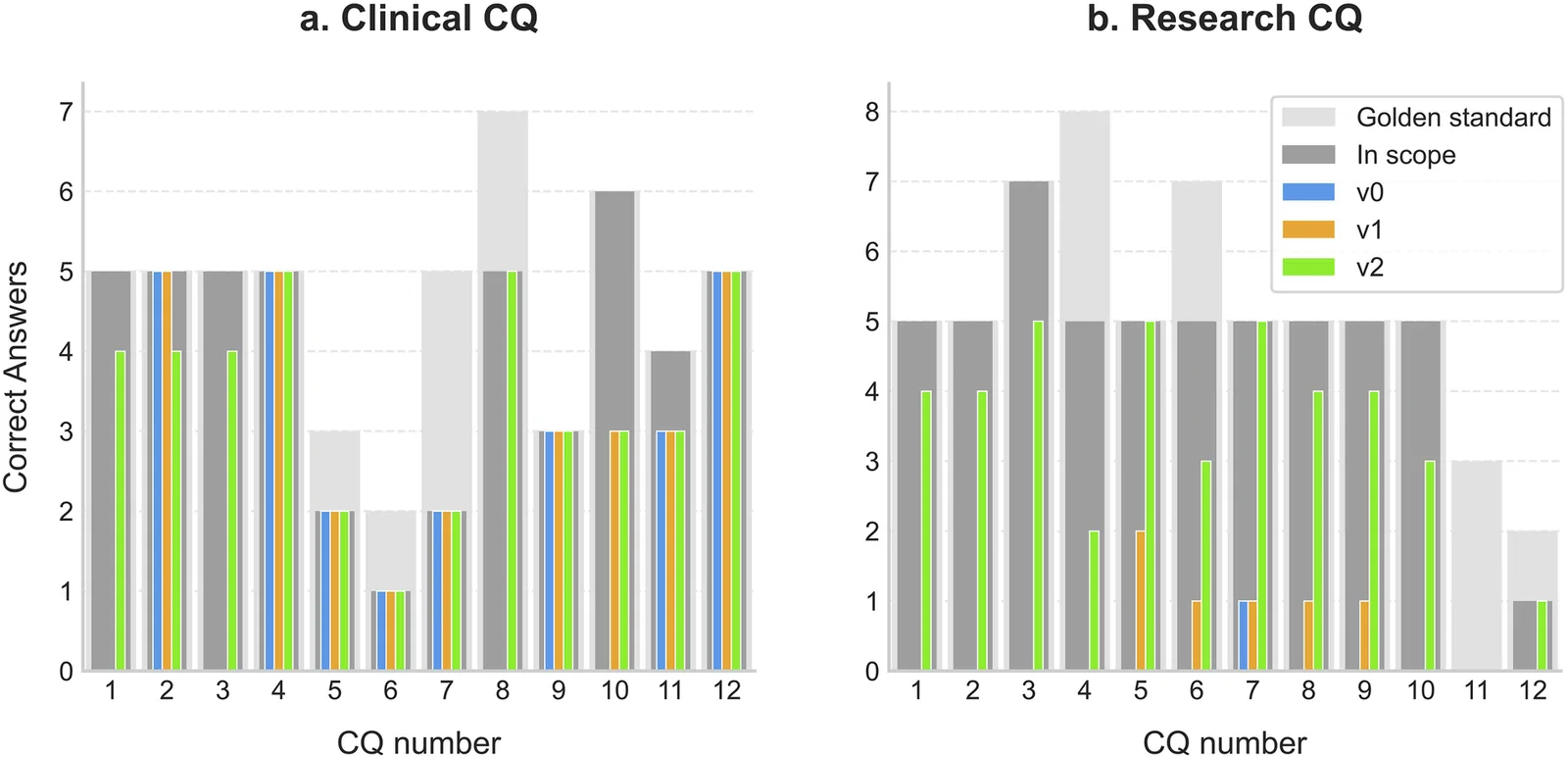
The CQ performance for 3 iterations of the DAKI-KG. *v0*: base version, *v1*: v0 enriched with SNOMED CT hierarchy, *v2*: v1 with grouped concepts.

Figure 5 shows the CQ performance of the DAKI-KG over different versions of the KG. The first version (v0) represents the base version, with solely directly linked SNOMED CT entities. The next version (v1) is extended with the full SNOMED CT hierarchy. The final version (v2) is the full DAKI-KG extended with nephrotoxic classes. Especially in the research queries, the inclusion of AKI and CKD classes has a notable impact. This impact is due to the higher number of AKI-related questions that benefit the most from this grouping, and less DDI-related questions, which do not require any grouping.

Overall, the clinical and the research application areas reached a recall of 0.75 and 0.65, respectively. When out-of-scope (OOS) errors were excluded, the recall reached 0.85 and 0.75 for the clinical case and the research case, respectively. This latter setting is the most appropriate to evaluate the KG quality, as it evaluates the KG only on information that was actually given, rather than expecting it to know information outside of its input. The overall recall (regardless of scope) is still meaningful, as this indicates which data should be added to future versions of the KG.

## Limitations and Final Remarks

The DAKI-KG provides structured insight into DDIs, side effects, indications and contra-indications, which are key concepts in the medication safety domain. Its novel contributions include distinguishing clinically actionable DDIs from those without apparent clinical impact, assigning frequencies to side effect annotations, and DAKI-specific data such as concept groupings and AKI risk factors. The DAKI-KG is therefore a valuable contribution to research, for areas such as variable/confounder identification, association discovery, question answering, and link prediction in the context of DAKI.

The current version of the DAKI-KG is not yet appropriate to use in a clinical setting, as the performance metrics show areas of improvement. Several limitations of the current DAKI-KG should be noted. First, the ELT used for mapping free text fragments about side-effects, contra-indications and indications from FK to SNOMED CT concepts, did not achieve perfect precision and recall, resulting in missing and sometimes misinterpreted terms and consequently incomplete representation of the source data. While a solid foundation is in place, finer-grained detail can be lacking.

Moreover, the SNOMED CT hierarchy lacks clearly defined levels of granularity that are generic across concepts. This complicates group-level queries, such as retrieving all kidney-related side effects, since related terms may be distributed unevenly across the SNOMED CT hierarchy. We demonstrated a hierarchical way of grouping in the evaluation, where we queried similar side effects between different drugs without predefined groupings. Note that this grouping can still miss terms when they are in completely different SNOMED CT branches. To mitigate this limitation, we defined disease-specific classes for AKI and CKD, which proved to be useful.

Nevertheless, while the DAKI-KG covers many diseases and drugs, its scope is centered on AKI. Consequently, we tailored such groupings, the risk factors, and CQs specifically to the AKI domain, and we cannot guarantee performance on a broader scope. In future versions, we plan to expand the DAKI-KG, for instance, by expanding the predefined groupings beyond kidney-related diseases, as well as investigating other approaches to groupings (e.g., text-based search). With this future expansion, we aim to investigate the MedDRA-to-SNOMED mappings specifically, as in the current setup, relevant terms may be overlooked or mapped to overly broad SNOMED categories that do not accurately reflect the intended group. This also applies to the AKI risk factors that we mapped to SNOMED CT identifiers; for example, the category *traumatic injury* includes specific sub-types that may not necessarily act as direct risk factors for AKI.

Furthermore, due to the time-consuming aspect of expert evaluation of the ELT, we currently evaluated a small sample, especially for the mappings representing contra-indications. We aim to extend this sample size in the future.

Finally, data extraction was limited to specific FK subsections. During evaluation, we found that sometimes information about contra-indications is presented under the warnings section. Therefore, the contra-indications included in the DAKI-KG are not complete.

## Data Availability

The DAKI-KG is made available on our *Zenodo* repository (https://doi.org/10.5281/zenodo.19661646). The repository includes all files that integrate into the DAKI-KG, except the full SNOMED CT hierarchy, and the SNOMED CT Preferred Terms: those require a license which must be obtained through the official SNOMED CT website (https://www.snomed.org/). Our GitHub (https://github.com/romy-vos/daki-kg) provides ready-to-use scripts to integrate the downloaded SNOMED CT data into the DAKI-KG.

## Code availability

The code for constructing and integrating the dataset, as well as the SPARQL queries used in our evaluation, can be accessed via our GitHub repository: (https://github.com/romy-vos/integration-daki-kg). This also includes the full list of kidney-related search terms used to search for nephrotoxic DDIs (data extraction/ddi/search_terms_nephrotoxic.json), and the input URLs used to retrieve data from Farmacotherapeutisch Kompas (data extraction/fk/extraction/urls). It also contains the code used to generate all plots presented in this paper. SPARQL queries and additional usage documentation can be found on (https://github.com/romy-vos/daki-kg).
